# Extra Supplement—The Nursing Mirror

**Published:** 1889-05-18

**Authors:** 


### The Nursing Mirror.

All Contributions for this Supplement should be addressed "The Nursing Mirror," care of the Editor, 139-140, Salisbury Court, Fleet Street, London, E.C.

### En Passant.

FITZROY HOUSE.—Mrs. Bluett is retiring from the post of matron of Fitzroy House, which she has held ever since the home was started eleven years ago. It is greatly owing to Mrs. Bluett's efforts that the pay hospital system has worked so well, and we shall be surprised if she does not get some united testimony from the leaders of the medical profession as to the value they place on her work. Mrs. Bluett has frequently had sixty, and sometimes upwards of a hundred, of the leading consultants to please in a year. Mrs. Bluett is going to be married, and Miss Young will reign at Fitzroy House in her stead.

SAD NEWS FROM INDIA.—It is with regret we hear of the death of Miss Baynes, better known in nursing circles in India as Sister Lucy. Some ten years ago Miss Baynes trained at the Clewer Sisterhood, and in 1881 was appointed superintendent of the Lady Canning Memorial Home in Calcutta. Here she trained many native women as nurses with great success. Miss Baynes had strong views on the respect due to native customs in India, and, therefore, was much liked by those under her. Miss Baynes started a convalescent home at Darjeeling, and aided many other similar efforts. For the last two years Miss Baynes suffered from a painful disease, which finally caused her death.

YOUTHFUL NURSES.—We do not like "Amicus." He writes to the *Chester Chronicle* on the subject of a more aged medical superintendent, and says: "Delicately nurtured girls are flocking in as nurses, and the natural anxiety of mothers and sisters cannot be wondered at. Happily, in our own institution they are cared for, and their welfare is anxiously studied by the devoted lady superintendent whose service the Board were able some years ago to secure. The comfort the knowledge of this fact must bring to many a home must be very considerable. If 'Nurse Josephine's Memories,' now appearing in a popular magazine, may be taken as an authority, things are occasionally so managed in some institutions as to be pleasant for neither nurses nor patients." A nurse's position is about the most trying a woman can be called upon to fill; but she has filled it for years to the glory of her sex and the improvement of her character. The man who, like "Amicus," fears otherwise, is lacking in chivalry and refinement.

SHORT ITEMS.—The bazaar in aid of Grimsby Hospital, organised by Dr. Damian, and managed by the nurses, brought in £100.—Mr. W. A. Nelson committed suicide last week during the absence of the nurse who had charge of him.—Miss Hampton, Miss Hampden, and Miss M'Dowel, trained nurses from the London, have all secured appointments at the Johns Hopkins Hospital, Baltimore, which we described last week.—"A Sympathiser" has written to the *Liverpool Courier* to point out that the nurses in the local infectious hospitals are only paid £30 to £35 a year. He asks if the city fathers could not afford to be more generous? What would "A Sympathiser" say to nurses in infectious hospitals who are only paid from £18 to £22? We know of many thus munificently remunerated.—Three district nurses are being trained at the Queen Victoria Institute, Edinburgh. The Jubilee Fund is being quickly made useful by our Northern sisters.—The annual gathering of the nurses of the Workhouse Infirmary Nursing Association will be held at the Grosvenor Gallery on the 20th of May, at 7 p.m.

MASSAGE.—There is a clearly-written and well-illustrated article on Mechano-Therapeutics in the "Medical Annual" which should be useful to those nurses who have studied this subject practically. The illustrations are specially good. How much massage is coming to the fore is shown by the prize-giving lately held at the West End Hospital, when there was quite a large gathering of ladies and nurses. The subjects a nurse is required to know are gradually increasing, and shortly we shall have from her the same cry of overwork which is at present heard from medical students.

WESTMINSTER NEWS.—The Duke of Westminster, whose interest in nursing matters is unfailing, presided last week at a meeting of the St. George's, Hanover-square, and Westminster branch of the Charity Organisation Society. The Duke pointed out the decrease of pauperism in the neighbourhood. Mrs. Garrett Anderson moved the following resolution: "That the Westminster Sanitary Aid and the Chelsea and Pimlico Nursing Associations, together with the convalescent arrangements of the Charity Organisation Society, are eminently calculated to benefit the poor; and those present give their hearty approval of the action of the St. George's Committee in carrying out these objects in the past, and pledge themselves to support these efforts in the future." She explained that sanitary aid was given by visits to people, and by checks to the spread of disease. As to nursing, skilled nurses were sent to poor people at their own homes. By these means she held that much good was being done. Dr. Bond, in seconding the resolution, referred to particular cases, showing that excellent work had been accomplished. The resolution was supported by Dr. Harrington Haward, Lady Frederick Cavendish, and Mr. C. S. Loch and carried.

AT THE ACADEMY.—Nurses on night duty cannot do better than spend a morning at the Royal Academy, where they can enjoy seeing many bright and beautiful pictures. Of course there are certain paintings which specially appeal to nurses, such as the portrait of Nurse Ann (675) by Macbeth-Raeburn. This represents a powerful-looking woman in the black cloak and bonnet which is now such a universal uniform; Nurse Ann belongs to the Middlesbro' Cottage Hospital. She is not the type of a sentimental nurse; we dare vouch she is a woman who works well. The portrait of Mr. T. Holmes (49) by Mr. Richmond will be interesting to the nurses of St. George's, and all will be glad to see the sweet face of the late Dr. Wilson Fox (1,231) as painted by Val Prinsep. The attempts at painting death all seem to miss the transparent waxy look with which a nurse is familiar; Marianne Stocks (358) gives very grey shadows on the face of a dead child, and Long's "Jairus's Daughter" (503) even seems to miss this look. "A Case for the Hospital" (198) is a clever sketch of a small child who has brought a broken doll into a carpenter's shop; "Flower Offerings" (1,223) is a church interior and village children bringing in great baskets of country blossoms, which are doubtless to be forwarded to hospital wards. In the Architectural Room is a drawing of the New Hospital for Women (1,916) now being erected in the Euston-road; 2,003 and 2,002 are also hospital designs. In the Central Hall is a marble bust of Dr. Dyce Brown (2,017), and one of Surgeon-General Gordon (2,043). In the Lecture Room are busts of the late Dr. Bruce Joy (2,124) and Prof. Robertson (2,154). After all, we expect nurses will prefer to note the many beautiful landscapes which are exhibited this year than to search out subjects which bear on their special interest. The Academy is not unpleasantly crowded now, except between the hours of three and five. The doors open at 8 a.m. and close at 7 p.m.

### Notes from Australia.

*(By Our Own Correspondent.)*

Melbourne, March 24.

This is a Sunday, and a most perfect morning. There has been a slight frost and a heavy dew; but in an hour the heat of the sun will have dried up all remembrance of the present freshness. With such surroundings one ought to do good work; but, alas! the very beauty of earth and sky tempts the thoughts away from hospital wards, and makes one long to simply exist out of doors.

It has recently been found necessary to remove certain lepers from the neighbourhood of Ballarat to the leper station at Point Nepean. The story of the proceedings is pathetic and pitiable. The poor wretches are rotting off the face of the earth, and our only duty to them is to ease their passage to death in every possible way; and to ourselves, to avoid by all means the slightest risk of infection by contagion. There does not appear to be much risk. We have had leprosy amongst us now for many years, and yet cannot point to one single case which would justify the conclusion that the disease may be acquired by contagion. But possibly we have been fortunate, or happily our climate and general environment do not produce the conditions necessary to the propagation of leprosy. A few weeks ago we were said to have only five cases of leprosy in the colony, but apparently they are now more numerous. Point Nepean is to the left side of the channel through which all vessels must come to Melbourne. It is a picturesque place, with a good view of all in-coming and out-going craft, but extremely lonely. Father Damien has done more to arouse interest in lepers, here as elsewhere, than any other man.

The Austin Hospital has not yet began the new consumptive wards so much wanted. It is strange that in a charitable place like Melbourne there is no consumptive home yet in existence, though it is greatly needed. The crowded condition of Melbourne Hospital calls for active measures. According to the report of the medical superintendent, it is so cramped for space that it is only fulfilling half of its duty to the public, and is even fulfilling that half imperfectly. It is not a question of supervision or management. The simple fact is that the hospital is admittedly too small to be effective. No patching up will be of any use; we must have a new and a thoroughly adequate hospital. At present there are seventy-six cases of typhoid in the hospital. Typhoid fever continues to be prevalent throughout the colony. The returns furnished to the Central Board of Health show that from December 1 to Saturday, February 16, over 1,150 cases had been reported, including 136 fatal cases. The disease has not been confined to a few isolated districts, but is widespread and general. The municipal authorities are of opinion, however, that typhoid fever has not been more virulent than it was twelve months ago, the increase in the number of cases reported being attributable to the fact that the reporting of cases was made compulsory by the Public Health Law Amendment Act passed last session.

The nurses at the Women's Hospital have been getting into disgrace; the fact being that there are not enough staff nurses and too many pupils. The medical staff have just issued the following report, which speaks for itself: "1. That the hospital is at present in a satisfactory sanitary condition. 2. The staff would draw attention to the large number of patients who are, and have long been, awaiting admission. At present they amount to 165 cases, and the number is steadily increasing, thus showing that the available accommodation—23 ordinary beds—is quite inadequate. 3. The staff would also refer to the insufficient number of special wards, of which there were only two, so that operations on patients who were dangerously ill had frequently to be deferred, to the greatly increased risk both to the patients themselves and the ultimate success of the institution. 4. They desire to point out that some of the nurses lately engaged are not sufficiently proficient in the special requirements necessary in a hospital of this nature, and they recommend that the nurses employed for special operation cases shall be permanently engaged in the hospital, in order that their experience may be increased, and a healthy competition for success encouraged amongst them.

The annual meeting of the District Nursing Society was held last week. The reports of the superintendence subcommittees show that the total number of patients on the society's books for the year was 363, of which 180 were discharged as cured or relieved, 22 removed to the hospitals, 2 to the Benevolent Asylum, 1 to the Convalescent Home, and 14 died. Owing to the want of funds during the past year, the committee were unable to carry out their resolution of either purchasing or renting a small house, centrally placed, where the nurses could reside, and where their meetings might be held, and to which donations from many charitable friends might be sent. This, of course, would require some alteration in the salaries of the nurses, and would increase in some way the expenses of the society, but it would render the work of the society more efficacious. The balance-sheet showed that the receipts for the year amounted to £513 7s. 4½d., which, added to the balance brought forward from last year, made a total of £584 0s. 7d. Out of this there remains to the credit of the society an unexpended balance of £263.

I hear from Adelaide that Miss Thackthwaite and Miss Burke, who were once sisters at the London Hospital, have both secured excellent posts, and are pleased with their reception in Australia.

There is a want felt by strangers in ill health who come to Melbourne. In England and Scotland the hydropathic institutions very much meet this want. Something not a boarding-house, lodging-house, or hotel. A kind of place where quiet, nursing, and good food are to be had on reasonable terms. Those living in Melbourne also would be only too thankful for a good refuge when health is failing, and the whirl of life and family cares is a little more than can be borne. At present there are two invalid homes in Melbourne. One, which is successfully carried on, was established in July, 1883, by Miss Macartney. A nurses' home was added to this in April, 1886, and is now doing very good work. Dr. Rowan has a private hospital, where many difficult cases from city and country find efficient treatment. The house is close to a busy part of Melbourne, but stands well in its own garden, with a good view of some reserve grounds and Government House. It is a short distance from Princes Bridge and Flinders-street railway stations. At Ballarat a private hospital is recently established in a central position, 179, Mair-street. Remembering my own experience at Ballarat, with a serious case of illness, and a very stuffy room in which to nurse my patient, I have no hesitation in saying this will be a boon to many.

### Nursing in the Soudan.

*(Continued from page xx.)*

Part VI.—The Victoria Hospital.

To enable our readers to picture the home and surroundings in which we passed three happy months of work, often arduous enough on account of the great heat of the Egyptian summer, I will endeavour to describe the hospital. It was situated about a mile and a half from the town of Suez, in the direction of the desert, and was built on four sides of a square, within which were thirty tents, with four beds in each. These latter were, however, taken down after we had been there some few weeks. The building which formed that side of the square facing the Sweet Water Canal was a hut containing beds for sick officers, the quarters of the sisters, the paymaster and sergeant-majors, the office of principal medical officer, and another hut for specially bad cases. On the right were the medical officers' quarters, on the left wards, on the remaining side of the square were two large wards, named respectively "Argyle" and "Northcote." The long, low, wooden huts were set on piles some 4ft. or 5ft. from the ground, being intended to allow of a free passage of air for the sake of coolness. The topmost roof came low down over a broad verandah on all sides of the huts, thus shading the wards from the burning sun. The lavatories were divided from the wards by a passage open at both ends. It must be remembered that drainage was a thing impossible, so that every precaution had to be taken to secure good sanitary conditions, a matter of vital importance in a climate where the thermometer had a playful way of running up to 90deg. and more in the shade. For some days 103deg. in the shade was recorded. The low military iron bedstead and straw mattress, with plain deal locker, tables, and forms pretty much completed the furniture of the wards, not forgetting the iron frame round the bedstead, supporting the indispensable mosquito curtains. This hospital was, we were told, originally built by the Indian Government, for the use of their sailors and others passing backwards and forwards through the Canal. This being so, in the garden round it were to be found many trees and plants brought from India and elsewhere. One tree had a magnificent crimson bell-shaped flower, which the shoeblacks had a fancy for, and used to put it to the odd use of blacking shoes. The juice from the crushed flower formed a sort of black dye, which had the effect of making boots black, if not shiny. The gardens were quite a feature of the hospital, and beautifully gay; such a rest to the eye from the glare of the sun on the endless expanse of desert surrounding it, The National Aid cooking hut, canteen stores, sisters' kitchens, and the like, formed a row of huts parallel with one side of the square, and were surrounded by palings, with an entrance, overlooked by the guard-house, where a sentry was on duty night and day. Outside, a short distance further in the desert, was a camp some 300 strong, where a company of the gallant Berkshire regiment was stationed.

The hours of work and general routine may perhaps be mentioned here. The sisters visited their wards in the morning at 7 a.m., sometimes earlier, according to the severity of their cases. At 8, breakfast, back to wards at 9, where all were fully occupied till 1, then luncheon was served. Two o'clock until 3 wards again, serving medicine and taking temperatures, etc. From 3 p.m. until 5, rest and tea. At that hour all were again in their wards until 7, when we dined, that being the only hour when a cool meal could be obtained with less chance of interruptions. At 8 p.m. a general round of wards for medicines, night temperatures, and special attention to bad cases before the night. The number of patients varied continually. At times work was very slack; then, again, a vessel from Suakim would bring us patients. On the 1st August, one hundred and twenty sick officers and men came in; men from the camp were also admitted as patients. Our cases were of various kinds—sunstroke, pneumonia, intermittent, typhoid, and rheumatic fevers. When the hospital was full of such bad cases it was often necessary for a sister to be on night duty as well as the orderlies; one out of four of the former was sadly missed from day duty. It was next to impossible to sleep in the heat of the day, so it often fell out that after a very slight rest she was to be seen in her ward as usual; needless to say that such continuous work soon told upon the physical strength, but this was only in busy times, and the orderlies were equally hard pressed. Had sleep been possible in the daytime, night was decidedly the pleasantest time for work, with an occasional amusing exception, to be narrated. The nights were not *always* moonlight, but the wonderful brilliancy of the stars in the Eastern sky prevented the black darkness so often to be seen at home. Nevertheless, unless the moon was visible, it was necessary to set out across the compound with a lantern when visiting at night from hut to hut, and these said lanterns were on more than one occasion our very good friends.

Alas! it was no fable, but a stern reality, that the wild dogs of the desert, who made night hideous with their howling, came inside the hospital square at dead of night, and sniffed in an embarrassing manner at the heels of the night sister. Now, a dozen or more of these long-legged, lean, hungry creatures may be harmless enough; but place yourself in imagination in such a situation, alone at night, and, if I mistake not, a certain foolish tremor will pervade you, and although the friendly lantern flashed in their faces would, for the time being, scare them off, it wasn't pleasant.

*(To be concluded.)*

### Lectures.

THE lecture last week at the club in Buckingham-street was delivered by Mr. Walter Pye, F.R.C.S., and dealt with the management of some slight deformities common in infancy. There was a good attendance of members, who listened with interest to Mr. Pye's very practical discourse, which contained many hints not found in ordinary text-books and manuals.

In examining an infant for the first time, the nurse should be sure to notice if it is in any way misshapen, as slight deformities can often be easily cured if attended to at once. The fingers may be webbed, for instance, or there may be supernumerary fingers or toes, in which cases the doctor should be informed immediately. Babies are always more or less club-footed; but if so much so that the feet will never right themselves, there can be no mistake about the deformity. There is no necessity to frighten the mother by mentioning the fact for a few days, as nothing can be done. Be very careful in handling a baby how you touch any lumps on the body, as rough treatment in cases of spina bifida may make the difference between life and death. In cases of hare-lip and cleft-palate there is no necessity to worry the mother for a day or two, as the third or fourth week is generally considered soon enough for an operation. It is difficult to feed such cases, but a clever nurse can do it very slowly and gently through some long funnel. If a child can arch its back when turned over with the nurse's hand beneath it, there is not likely to be much wrong with it. Ill-nourished children cannot keep the concave position after six months; their spines are unduly flexible. A wobbly spine is an early sign that a child is not properly nourished, and generally indicates rickets. If a baby has acute wasting rickets, and the nurse handles it carelessly, she may cause all the limbs to curve; but by judicious handling and rubbing these deformities can be greatly lessened. A limb is badly nourished; rub it gently with the tender palm of the hand from the extremity upwards; you can call it massage if you like, though the massage of infants is nothing more nor less than an infantile proceeding. Infantile paralysis, coming on suddenly with a fit, leaves one limb paralysed; keep that limb warm and rub it constantly, keeping it in a natural position; then, when power returns the limb will be useful and in no way deformed. When children insist on walking too early, it is no use to tie them in bed, or their health will suffer; put them on straight splints, well-padded and firmly bandaged on, these will hobble them sufficiently to prevent bandy legs.

The lecture was loudly applauded, and several members took advantage of the occasion to question Mr. Pye on certain subjects of child management.

### Everybody's Opinion.

*Correspondence on all subjects is invited. No communications can be entertained if the name and address of the correspondent is not given, or unless one side of the paper only be written on.]*

### THE BRITISH NURSES' ASSOCIATION.

A HOSPITAL CERTIFICATED NURSE writes: We are hearing a great deal now about this much-vaunted British Nurses' Association. Of course, most of what we have heard has been from its supporters, and therefore decidedly in its favour, for most of us nurses do not trouble ourselves about it one way or another, but let things take their own course. Now, however, I for one think it high time no longer idly to take it for granted that the British Nurses' Association is a perfect godsend to all nurses, but to consider carefully what this association professes to be able to accomplish, how it sets to work to carry out its proposals, whether it really will be of use to us nurses, and finally whether it will be well for one and all of us to join it without further delay.

For this purpose I will take seriatim the various statements made in a pamphlet issued by this association. This pamphlet states that the British Nurses' Association has been founded "to unite all British nurses for their mutual help and protection, and for the advancement in every way of their professional work. . . . Every person who claims to be a nurse, who engages in the work of nursing, without having been thoroughly prepared for the duties which that work involves, is likely to diminish not only the demand for them, but also the payment which they will be entitled to receive," and, moreover, it "aims, in the first instance, at bringing about a state of things in which no one will be allowed to call herself a nurse unless she has undergone a proper course of hospital training, of a sufficient length to give her an insight into the management of all kinds of illness, whether surgical or medical." So far so good, and if this association were careful to receive only those nurses who can bring proper guarantees of thorough training, it might carry out its professions. And how does the British Nurses' Association set to work to attain this very desirable object, and endeavour to protect the unfortunate public, whom one would imagine all nurses had hitherto combined to injure? And why, moreover, should the public be excluded from belonging to the association? Is the association afraid that the public will rush in in such large numbers as to be inconvenient?

To return, the association will endeavour to secure properly trained and efficient nurses to the public by allowing that "every woman actually engaged in nursing is eligible," though just before it has been stated that "at present, when we hear that a woman is a 'nurse,' we hardly know what meaning to attach to the word; but when the association has succeeded in the attainment of its primary object, this meaning will be no longer doubtful." Here we have an association proposing to protect the public against unqualified nurses, and stating "that the British Nurses' Association guarantees that the person to whom this word 'nurse' is applied has passed through a prescribed course of study and practice, and has given satisfactory proof of having turned her opportunities of learning to good account," and, on the other hand, welcoming, as a member, anyone who has been engaged in nursing for three years, thus implying that any "Sairey Gamp" may have what the promoters look upon evidently as a much-to-be-coveted honour. Would anyone who has had anything to do practically with nurses make such an absurd blunder? And suppose that hospital nurses who have toiled through their two or three years' training, and finally obtained their precious certificate, and by their conscientious work have won the respect and goodwill of those in authority over them—will such wished to be mixed up in a sort of "hotch-potch" with the scum of the nursing profession? Anyone who knows anything about nursing will tell the promoters of this association that it is only quite recently that workhouses and asylums have discovered the necessity for trained nurses, and that, however zealous the authorities of these excellent institutions are, they can only work reforms slowly, and that there are many of the old order of nurses left. Nevertheless, the Benevolent British Nurses' Association demands only that all would-be members should have been engaged, in whatever capacity, in nursing for three years.

The worst of it is, that this sort of association will probably attract a large number of nurses from country hospitals, who have not the advantage of being connected with a hospital in London, and hearing nursing matters discussed, and both sides stated; so that they can have all the *pros* and *cons* of the cases before them Perhaps, too late, nurses will realise that the public and the authorities of the large training schools will indeed "know what meaning to attach to the words member of the British Nurses' Association"—namely, a nurse who has taken refuge in it to obtain pseudo-respectability, because she could not get it elsewhere. What, then, does the British Nurses' Association offer its members in return for their half-crowns? A so-called certificate with a facsimile of Her Royal Highness Princess Christian's signature! And what use is this certificate to its owner? We will hope the founders of the British Nurses' Association can answer this question—no one else can! To hospital nurses it appears a mere farce—about as useful as the "V.R." over a letter-box—nay, less so, because Her Majesty's initials do guarantee that the letter-box is a properly-authorised deposit for letters, and that a properly-appointed officer will be responsible for all letters thrown into it. But does Her Royal Highness Princess Christian also guarantee that every nurse possessing the paper with a facsimile of her signature is also a properly-qualified member of the great nursing profession, and are those who presume to give the certificate properly qualified to judge whether she deserves it or not? We absolutely deny that a self-constituted association like the British Nurses' Association is fit to grant, from second-hand information, a certificate to any nurse who applies for it, and can pay her half-crown. Truly, if this is so, and this passport can be so easily obtained, there is no more call for the careful and systematic training given to their nurses by the large hospitals. The British Nurses' Association will kindly relieve them of all trouble and responsibility. Nurses, after three years' nursing in whatever capacity, have only to pay down their money and they can defiantly wave the magic certificates before the face of a helpless public. Everyone knows how many women apply at hospitals to be trained as probationers, what a number of these fail or have to be dismissed as unsuitable before the end of their training, but because they have been in a hospital for a short time they think they can be private nurses. Such are the women who, we venture to prophesy, will rush into such an association. And we ask any impartial judge, are such women calculated to raise the status of the nursing profession? If such people make up the members of which the British Nurses' Association announces itself to be composed, we wish it joy of its members. And far from regretting, as the pamphlet does, that the authorities of a few public institutions have advised their nurses not to join the British Nurses' Association; we, with many other hospital-trained nurses, regret very much to hear that any institutions have shown so little regard for their nurses' true welfare as not to have used every effort in their power to prevent their joining so hurtful an association. The British Nurses' Association has evidently been formed to drag the nursing profession into a prominence not desired by the best nurses, who treasure most the respec and goodwill of their *alma mater*, where their powers are truly appreciated.

### A HINT TO MIDWIVES.

MRS. BEDINGFELD writes: Seeing your note in the current number, entitled "A Hint to Midwives," I venture to tell you a plan I adopt with success. I lay no claim to originality, but it may be new to some of your readers. I wipe all discharge from the baby's eyes, with a clean soft cloth, as soon as ever it is possible. Then, when proceeding to wash it, I take a piece of soft clean linen rag, and carefully wash the eyes with warm water till they are scrupulously clean, letting the clear water trickle over them. I also wash the mouth out with a piece of rag, and wipe the face dry, before continuing with the bath. When bathing the child, I am most careful that not a drop of the water in which the child's body has been washed touches the eyes. This is the point I specially wish to emphasise. I repeat this process every morning, using a fresh piece of linen each time, until the child is at least a fortnight old, never using for the face, eyes, or mouth either the sponge or flannel used for the body. When attending the poor, I teach them to take the same precautions. The result is that babies under my care rarely (I might almost say never) have either bad eyes or thrush. And in cases in which there is hereditary taint, causing, with all one's care, a certain amount of eye mischief, the evil by my plan is greatly mitigated.

### Vacancies.

The following vacancies are announced:

**Nurses.**

West London Hospital, £24.

Stockford Infirmary, £24.

City of London Hospital, Victoria Park, £22.

Nurses' Institute, Jersey, £25.

Bradford Infirmary, £25.

Nurses' Institute, Bedford.

Ashburton Cottage Hospital, £25.

North London Nursing Institute.

Grimsby Hospital, £25.

Dumfries Infirmary, £20.

Hartlepool Union, £25.

Eastbourne Nursing Institution.

Nurses' Home, Frome.

Workhouse Infirmary Nursing Association.

Newlands, Ryde.

Notts. Nursing Institution, £25.

Nursing Institute, Ryde.

Brecknock Infirmary, £20.

Carmarthen Infirmary, £23.

Royal Albert Hospital, Devonport (several), £25.

Darent Asylum (head), £30.

Peterborough District Nursing Association, £30.

Birmingham Fever Hospital, £20.

**District Nurses.**

Daresbury, Warrington.

4, Gresham-place, Newcastle-on-Tyne, £40.

Newport, Isle of Wight, £30.

Bedford Nurses' Institute.

**Probationers.**

Grimsby Hospital.

Burton-on-Trent Institution.

Temperance Hospital, Hampstead.

Bristol Hospital for Sick Children

Lancaster Infirmary.

### Notes and Queries.

**Queries.**

(14) *Homes of Rest.*—Where can a governess, out of health, be taken for three weeks' change and care? *B.*

(15) *Free Home for Nurses.*—Is there a convalescent home where a nurse would be taken free? *Bournemouth.*

**Answers.**

(9) *Accoucheuse.*—You had better apply to the Secretary, Midwives Institute, 15, Buckingham-street, Strand. That is the most likely place to hear of such an opening as you require. It is no use to go to Canada unless you have good introductions, or friends there. *Colonist.*

(11) *Nursing in Asylums.*—The Berry Wood Asylum, Northampton, is advertising for a nurse for the infirmary. Watch our advertising columns. *Editor.*

(13) *Nurses as Stewardesses.*—We regret to inform *West Ham* that the register is closed and her name cannot be entered.

(14) *Homes of Rest.*—Apply to St. Martin's Lodge, Scarborough (15s. a week), Catherine House, Hastings (21s. a week), or Hartington House, Buxton (15s. a week).

(15) *Free Home for Nurses.*—Mrs. Marshman's Home, at Brighton, is free by letter from subscriber, so is the Convalescent Home at Cookeridge and Mrs. Gladstone's Home at Woodford. If none of these will do, apply to Mrs. H. Pease, Darlington, or Mrs. Tudor Owen, Rhyl, Wales.

(8) *Nursing in America.*—I see A. wishes to know about nursing in America, and as I was there last year I can give her some addresses: New York Hospital, 15th-street, New York City; St. Luke's Hospital, New York City; Brooklyn City Hospital, Brooklyn, Long Island, U.S.A.; Long Island College Hospital, Henry-street, Brooklyn. They have always a large number of applicants for their vacancies. *E.M.S.*

